# Blood culture contamination in a tertiary care hospital: a retrospective three-year study

**DOI:** 10.1186/s12879-023-08428-0

**Published:** 2023-07-04

**Authors:** Banan M. Aiesh, Duha Daraghmeh, Nasreen Abu-Shamleh, Abdalmenem Joudallah, Ali Sabateen, Rowa’ Al Ramahi

**Affiliations:** 1 Infection Control Department, An-Najah National University Hospital, Nablus, Palestine; 2 Department of Pharmacy, Faculty of Medicine and Health Sciences, An-Najah National University, Nablus, Palestine

**Keywords:** Blood culture contamination, Palestine, *Staphylococcus epidermidis*, Tertiary care hospital

## Abstract

**Background:**

Bloodstream infections (BSI) are a leading cause of morbidity and mortality in hospitalized patients worldwide. A blood culture is the primary tool for determining whether a patient has BSI and requires antimicrobial therapy, but it can result in an inappropriate outcome if the isolated microorganisms are deemed contaminants from the skin. Despite the development of medical equipment and technology, there is still a percentage of blood culture contamination. The aims of this study were to detect the blood culture contamination (BCC) rate in a tertiary care hospital in Palestine and to identify the departments with the highest rates along with the microorganisms isolated from the contaminated blood samples.

**Method:**

Blood cultures that were taken at An-Najah National University Hospital between January 2019 and December 2021 were evaluated retrospectively. Positive blood cultures were classified as either true positives or false positives based on laboratory results and clinical pictures. Statistical analysis was performed using the Statistical Package for Social Sciences (SPSS) version 21. A p-value of less than 0.05 was considered statistically significant for all analyses.

**Results:**

Out of 10,930 blood cultures performed in the microbiology laboratory from 2019 to 2021, 1479 (13.6%) were identified as positive blood cultures that showed microbial growth. Of these, 453 were blood culture contaminations, representing 4.17% of total blood cultures and 30.63% of the positive blood culture samples. The highest rate of contamination was in the hemodialysis unit (26.49%), followed by the emergency department (15.89%). *Staphylococcus epidermidis* was the most prevalent (49.2%), followed by *Staphylococcus hominis* (20.8%) and *Staphylococcus haemolyticus* (13.2%). The highest annual contamination rate was observed in 2019 (4.78%) followed by 2020 (3.95%) and the lowest was in 2021 (3.79%). The rate of BCC was decreasing, although it did not reach statistically significant levels (P value = 0.085).

**Conclusion:**

The rate of BCC is higher than recommended. The rates of BCC are different in different wards and over time. Continuous monitoring and performance improvement projects are needed to minimize blood culture contamination and unnecessary antibiotic use.

## Introduction

Bloodstream infections (BSIs) including fungemia and bacteremia, are a leading cause of morbidity and mortality in hospitalized patients worldwide [1]. Obtaining a blood culture is the primary tool for determining whether a patient has a BSI and requires antimicrobial therapy, but it can produce incorrect results in which the reported microorganism(s) are deemed to be contaminants. Despite advances in medical equipment and technology, there is still some blood culture contamination (BCC) [2]. It is very important to distinguish between a true BSI and a contaminated blood culture to avoid unnecessary use of antibiotics, a longer hospital stay, and extra costs [3, 4]. Based on the recommendations of the Clinical and Laboratory Standards Institute (CLSI), the acceptable rate of BCC in health institutions is less than 3% [2, 5]. Microorganisms may gain access to the bloodstream through the patients’ skin, their contaminated surrounding, the contaminated hands of health care providers and the equipment used for blood sampling or transfer [2]. The most common microorganisms that cause BCC were reported to be coagulase-negative *staphylococci, Micrococcus* species, *Propionibacterium* species, *Bacillus* species, and *Corynebacterium* [1, 6].

The causes of contamination may be inappropriate sampling, low staff experience in processing or transporting the blood or blood product, non-compliance with aseptic techniques, high traffic and overcrowding in the areas of blood collection, or difficulty in drawing blood from children and elderly patients [7]. To determine whether the culture is a true positive or contaminated, clinicians will judge based on the symptoms of the patient, elevated infection markers such as leucopenia, the number of positive blood culture sets, the time to positivity, the presence of an indwelling catheter during hospitalization, and some microbes that rarely cause blood stream infections [8].

The techniques for collecting and testing samples are critical. In our hospital, the patient’s injection site is disinfected with an alcohol swab by the nurses, and then blood sampling is performed by inserting the needle after repeatedly palpating the area with gloved hands to check for prominent veins. The clinical microbiology laboratory acts as an essential part of providing key data to healthcare workers when the result of a blood culture is possibly contaminated. In our hospital, blood samples are inoculated into VersaTrek Redox 1 aerobic and Redox 2 anaerobic media (Thermo Fisher Scientific, Waltham, MA) and evaluated using the VersaTREKTM automated microbial detection system (TREK Diagnostic Systems, Cleveland, OH, USA). Incubation is continued for up to 5 days or until a positive culture is observed. Positive bacterial cultures are examined for pathogen type and antibiotic sensitivity. VITEK 2® COMPACT (bioMérieux, Marcy-I’Étoile, France) is used to identify bacteria and their antibiotic sensitivity.

The BCC rate is usually monitored in hospital settings because a low rate of BCC is a key indicator of laboratory and nursing quality. A contamination rate threshold is to be defined and the sources of contamination should be tracked. Blood culture is the best diagnostic test for sepsis, but the occurrence of a false positive blood culture which may result from blood contamination, due to the normal flora of the skin or other bacteria appearing in the sample, makes it less effective and leads to the consumption of unnecessary antibiotics that can result in increased antimicrobial resistance, increased cost and longer stays in hospitals [9].

The aims of this study were to monitor the contamination rate of blood culture samples in a tertiary hospital in Palestine and to identify the departments with the highest rates along with the microorganisms isolated from the blood samples.

## Methods

### Study design and setting

The study was a retrospective examination of blood culture results performed at An-Najah National University Hospital (NNUH) between January 2019 and December 2021. This healthcare institution provides wide medical and surgical services through intensive care units (ICUs), medical, oncology, surgery, hemodialysis, emergency, pediatric, vascular, bone marrow transplant and cardiology departments, in addition to outpatient clinics.

### Ethical consideration

All relevant parts of the study, in the form of patients’ clinical data and access to this data, were reviewed and approved by the *Institutional Review Board* (IRB) of An-Najah National University. There was no need for patients’ informed consent because the study was based on culture data that had already been examined in the laboratory. So informed consent was waived by the IRB. We had confirmed that the collected information was only used for clinical research purposes and that access was limited to study group staff. The identities of the patients were not shared, and we used numbers to code the cases.

### Study population

The targeted population of this study was all patients from An-Najah National University Hospital departments who had a positive contaminated blood culture sample. The inclusion criteria were as follows: [1] all patients with blood culture contamination; [2] patients from all hospital departments; [3] patients with all comorbidities and clinical diagnoses. Exclusion criteria included patients with true positive blood cultures.

### Sample size

We studied all patients who were admitted to any hospital department between January 2019 and December 2021, who were classified as having contaminated blood culture. The overall sample size that met the inclusion criteria was 453 samples.

### Criteria to determine contamination

The clues and criteria that were followed to distinguish contamination from true infection were: [1] the identity of the isolated microorganism (e.g. coagulase-negative staphylococcus spp. *Corynebacterium*, *Bacillus* spp. other than *Bacillus anthracis*, *Micrococcus* spp, *Propionibacterium acnes*) [2]. the number of positive cultures. These organisms were not considered contamination if they were isolated from at least two sets of blood samples [3] patient’s clinical status, history and laboratory findings such as fever, leukocytosis or leukopenia, and high C-reactive protein [10]. In summary, contaminations were determined based on the number of positive blood cultures and the organism’s identity; for example, a culture was deemed to be contaminated if one of the two sets of blood cultures showed a skin commensal pathogen [11] as well as the patient’s clinical scenario. The samples were evaluated by an Infectious Disease (ID) clinical pharmacist from the infection control unit.

The blood culture contamination rate (percentage) was calculated by multiplying the number of contaminated blood cultures by 100 and dividing it by the total number of routine blood cultures obtained [7].

### Data collection instrument

Over the course of this three-year study, the entire number of blood cultures processed from various wards, as well as the total number of infected blood cultures and cultures that showed no growth, were calculated and arranged in tables using Excel sheets. Routine procedures were used to identify positive blood cultures, which were classified as either real bacteremia with a confirmed bloodstream infection or blood culture contamination (false positive). The information about the patients including age, gender, wards, and culture findings, was collected from electronic medical records.

### Data analysis

IBM SPSS Statistics for Windows, version 21 was used to conduct the data analysis (IBM Corp., Armonk, N.Y., USA). A descriptive analysis was carried out for demographic, clinical characteristics, and causative microbial organisms. Categorical variables were presented as frequencies and percentages. Mean ± standard deviation was computed for continuous data. Categorical variables were compared using the Chi-square test or Fisher test as appropriate. A p-value of less than 0.05 was considered statistically significant for all analyses.

## Results

### Demographics and overall rate of blood culture contamination

Out of 10,930 blood cultures performed in the microbiology laboratory from 2019 to 2021, it was found that 1479 (13.5%) samples were positive blood cultures that showed microbial growth, while the rest of the cultures showed no growth and were negative blood cultures. Of these 1479 positive cultures, 453 were blood culture contaminations, representing 4.1% of total blood cultures and 30.63% of the positive blood culture samples. The age group that contributed to the highest number of contaminated samples was 60–79 years with 32.9% of the overall contaminated samples as shown in Table 1.

**Table 1:** Blood culture contamination rates in different age groups

Parameter	BCC frequency	BCC (%)
Age (years)		
0–19	103	22.7
20–39	66	14.6
40–59	121	26.7
60–79	149	32.9
≥ 80	14	3.1
**Total**	**453**	**100**

Comparing the departments to each other regarding the BCC rate, the highest contamination rate was found to be in the hemodialysis unit (26.5%), followed by the emergency department (15.9%). The lowest rate was in the pediatric intensive care unit, representing 3.5% of the total samples as detailed in Table 2. The contaminated blood samples were studied regardless whether they were from central or peripheral venous catheters. It was found that the rates were almost the same between the two sites, with the peripheral venous blood samples accounting for 50.9% of the total BCC.

**Table 2:** Frequency and percentage of blood culture contamination (BCC) in different wards

Wards	BCC (frequency)	BCC (%)
Hemodialysis	120	26.49
Emergency	72	15.89
Surgery	49	10.82
Medical intensive care unit	46	10.15
Surgical intensive care unit	38	8.39
Pediatrics	33	7.28
Internal medicine	23	5.08
Oncology clinics	19	4.19
Bone marrow transplant unit	19	4.19
Cardiac care unit	18	3.97
Pediatric intensive care unit	16	3.53
**Total**	**453**	**100**

### Causative microorganisms of BCC

The causative pathogens for BCC were studied, which resulted in *S. epidermidis* being the most prevalent species (49.2%), followed by *S. homini*s which accounted for 20.8% of the isolated pathogens, and then *S. haemolyticus* (13.2%). Other species that were identified are shown in Table 3.

**Table 3:** Distribution of isolated microorganisms as BCC

Microorganism	Freq.	(%)
*S. epidermidis*	223	49.2
* S. hominis*	94	20.8
* S. haemolyticus*	60	13.2
*Corynobacterium* spp.	28	6.2
*Bacillus* spp.	20	4.4
* S. capitis*	14	3.1
* S. warneri*	7	1.5
* S. saprophyticus*	5	1.1
* S. caprae*	1	0.2
*Kocuria*	1	0.2
Total	453	100

### Distribution of contamination rates in the three years

The contamination rates were different over the years. As shown in Table 4, the highest annual contamination rate was observed in 2019 (4.78%), followed by 2020 (3.95%), and the lowest was in 2021 (3.79%). The rate of BCC was trending down over the three years, but this reduction was not statistically significant *(p-value* = 0.085).

**Table 4:** Blood contamination rates in the three years

Year	Total number of blood cultures	Number of Positive cultures	Rate of positive results (%)	True positive	True positive (%)	False positive	False positive (%)
2021	4,082	542	13.27	387	9.48	155	3.79
2020	3,565	484	13.57	343	9.62	141	3.95
2019	3,283	453	13.79	296	9.02	157	4.78

## Discussion

Blood culture is still the most reliable laboratory diagnostic test for bloodstream infections, despite not being the most sensitive method. However, false positive blood cultures, also known as blood culture contamination, can frustrate physicians and microbiologists. The doctors rely on certain risk factors and clinical manifestations to distinguish contamination from positive blood cultures.

There were few studies that assessed the prevalence and pattern of sensitivity of bloodstream infections in our institution. One of them was a 2-year study among hemodialysis patients, which concluded that 99 (83.89%) of the isolated blood culture pathogens were gram-positive and 19 (16.1%) were gram-negative [12]. Another study in our institution among solid tumor patients revealed that the most common source of positive blood cultures was catheter-related. Gram-positive bacteria accounted for 52.6% of blood cultures, with a predominance of Staphylococcus species. On the contrary, gram-negative bacteria were documented in 39.7% of the cultures, with E. coli being the most frequent bacteria [13].

In this study, *S. epidermidis* was the most frequent contaminant (49.2%). This is in line with a Saudi Arabian study where *S. epidermidis* was found to be the primary cause of BCC [2]. S. epidermidis is the most frequently encountered member of the coagulase-negative staphylococci on human skin, which could explain this finding. However, it is an opportunistic pathogen and can become virulent when it invades the human body via indwelling medical and prosthetic devices, so it is very important to distinguish BCC from real positive culture. Other causative pathogens in our study included *S. hominis* (20.8%), *S. haemolyticus* (13.2%), and Corynebacterium species (6.2%). Skin normal flora, such as Coagulase-negative *Staphylococcus*, *Micrococcus*, *Alpha-hemolytic viridans* group, *Bacillus* spp., *Propionibacterium acne* and *Corynebacterium* spp. have been reported in earlier studies as the most possible culture contaminants [1, 7, 11, 14].

In recent years, an issue that is actually complicating the judgment regarding true bacteremia versus contamination is the increasing utilization of indwelling vascular access devices (VAD). The interpretation of culture results for patients with central venous access is challenging as these patients are at increased risk for bacteremia, while the isolated pathogens can also indicate culture contamination or colonization of the line [15]. The majority of contaminated blood cultures in this study were received from hemodialysis unit at a rate of 26.5% compared with an Indian study in which the emergency department had the highest rate of 54% [16]. A South African study conducted by Opperman et al. in 2020 showed that 13.4% of the BCC were from the emergency and trauma units [17] and 10.3% from the medical ward as Hemeg HA et al. declared [2]. Emergency departments are highly susceptible to an increased burden of contaminated blood cultures due to the acuity of cases that need blood culture samples upon resuscitation, high staff turnover, and the time pressure of obtaining cultures before the first dose of antibiotics [18]. Regarding emergency departemnt in our study, the overall contamination rate was 15.89% of the total samples, so improvement projects and education of staff using a blood culture collection list and monthly feedback can reduce the BCC rate.

BCC was most prevalent in the age group of 60–97 years in our study, with a rate of 32.9%. This was consistent with previous studies, which revealed that the highest rate of contamination was among the elderly (60–80 years) [2].

The annual BCC contamination decreased over the three years, reaching its lowest level in 2021 with 3.79%. Despite this, the reduction was not statistically significant. Noting that this rate is above the global benchmark of 3%. A study conducted in a large academic hospital in Europe resulted in an overall contamination rate of 6.3% [19]. This could be attributed to the fact that our institution is a tertiary academic center and that the blood culture contamination was not a targeted performance indicator.

Blood culture contamination has been linked to a number of factors, including unhygienic and improper aseptic techniques used when drawing blood, particularly by untrained staff, so trained and competent nurses result in fewer BCC. It has been observed that using alcoholic solution before taking blood samples will lower the rate of contamination [8]. As it cleans the upper dermal layers of skin and eliminates the majority of the commensal bacterial load, the back-and-forth friction method of applying the disinfectant prior to blood taking has been observed to be more effective in minimizing contamination than other approaches [20]. Insufficient blood volumes for culture are additional factors that negatively affect the contamination rate [7]. In this study, the patient’s injection site was disinfected with an alcohol swab and then blood sampling was performed by inserting the needle after repeatedly palpating the area with gloved hands to check for prominent veins. Inadequate aseptic practices by not waiting for the recommended contact or drying time of the antiseptic solution prior to phlebotomy could be a contributing factor to the higher-than-desired BCC rate in this study. Other factors could be unintentional errors in collection methodologies, the limited experience of the nurses, and the high workload.

A high BCC rate was seen in the hemodialysis unit. This unit is very crowded and includes many machines, which may increase the risk of contamination. Many of the patients were elderly with concomitant conditions, it was challenging to take blood from them, which may have contributed to a high BCC rate in the ICU and general ward as well. Patients with end-stage renal disease (ESRD) and older patients were more likely to be associated with BCC in another study [21].

The BCC decreased from year to year (from 2019 to 2021), this may be attributed to the awareness that was initiated by the infectious disease team regarding BCC, reporting BCC in a timely manner to relevant staff and education through the nursing continuous education program on proper technique of blood culture sampling. These interventions were introduced in January 2021 and thereafter on a continuous basis. During the Coronavirus disease 2019 (COVID-19) pandemic, adherence to infection control measures in terms of hand hygiene, antisepsis and personal protective equipment could have impacted this reduction in BCC.

We deduce from this study that efforts should focuse on having an adequate staff of trained nurses and adopting proper aseptic procedures with professional supervision to prevent BCC when the department is crowded with visitors and patients.

Our study suggests that by focusing on staff training, efforts should be made to reduce the elevated false positive blood culture rates to an acceptable level (3%). The nurses should be educated and committed to adhering to sterile and aseptic procedures during blood culture collection. This was effective in other studies [22–24]. Before administering an injection, the skin should be thoroughly disinfected and completely allowed to air dry. It is important to fully disinfect the rubber diaphragm on blood culture container tops and the catheter diaphragm on intravascular devices using 70% ethanol or isopropyl alcohol [25].

Efforts to reduce BCC rates could shorten the length of hospital stays and the use of unneeded antibiotics, which would save costs and lessen the danger of the emergence and growth of drug resistance. The BCC rates at our targeted tertiary care and academic hospital had never been examined before. All hospitals should routinely monitor the blood culture contamination rate and aim for a rate of ≤ 3% [25]. This would lessen the establishment of drug-resistant strains, lower contamination rates, shorten hospital stays, and hence lessen the financial burden.

Despite the fact that our study was the first to describe BCC in our country, including different wards and data from a three-year period, it still has some limitations, such as the fact that it is a descriptive, single-center study, and in addition, we didn’t study the sensitivity pattern of the isolated pathogens from the contaminated cultures. So, other comparative studies with larger samples that involve multicenters are recommended. Also, the true positive cultures were not evaluated and followed up to give a bigger picture of bacteremia/septicemia in our setting. It would be very useful to evaluate this in future studies.

### Conclusion and recommendations

The rate of BCC is higher than recommended. The BCC rate is different in the different wards and over the years, ensuing regular monitoring and performance improvement in a standard way over time. To stop contaminants from slipping into the blood culture, appropriate and routine blood culture intervention methods should be integrated into all patient care settings. Routine surveillance of BCC rates would highlight the current situation. The goal should be to reduce BCC in hospitals by enhancing proper sterility measures, strengthening sample practices, and hiring qualified and committed nurses. In order to identify false positives and get rid of the negative consequences that follow, clinical indications and symptoms should also be taken into account while analyzing laboratory findings from blood cultures, preferably in consultation with the infection control team.

## Data Availability

The datasets used and/or analysed during the current study are available from the corresponding author on reasonable request.
